# Integrating geometries of ReLU feedforward neural networks

**DOI:** 10.3389/fdata.2023.1274831

**Published:** 2023-11-14

**Authors:** Yajing Liu, Turgay Caglar, Christopher Peterson, Michael Kirby

**Affiliations:** ^1^Department of Mathematics, Colorado State University, Fort Collins, CO, United States; ^2^Department of Computer Science, Colorado State University, Fort Collins, CO, United States

**Keywords:** ReLU feedforward neural networks, binary vectors, polyhedral decomposition, geometries, Chebyshev center, Hamming distance

## Abstract

This paper investigates the integration of multiple geometries present within a ReLU-based neural network. A ReLU neural network determines a piecewise affine linear continuous map, *M*, from an input space ℝ^*m*^ to an output space ℝ^*n*^. The piecewise behavior corresponds to a polyhedral decomposition of ℝ^*m*^. Each polyhedron in the decomposition can be labeled with a binary vector (whose length equals the number of ReLU nodes in the network) and with an affine linear function (which agrees with *M* when restricted to points in the polyhedron). We develop a toolbox that calculates the binary vector for a polyhedra containing a given data point with respect to a given ReLU FFNN. We utilize this binary vector to derive bounding facets for the corresponding polyhedron, extraction of “active” bits within the binary vector, enumeration of neighboring binary vectors, and visualization of the polyhedral decomposition (Python code is available at https://github.com/cglrtrgy/GoL_Toolbox). Polyhedra in the polyhedral decomposition of ℝ^*m*^ are neighbors if they share a facet. Binary vectors for neighboring polyhedra differ in exactly 1 bit. Using the toolbox, we analyze the Hamming distance between the binary vectors for polyhedra containing points from adversarial/nonadversarial datasets revealing distinct geometric properties. A bisection method is employed to identify sample points with a Hamming distance of 1 along the shortest Euclidean distance path, facilitating the analysis of local geometric interplay between Euclidean geometry and the polyhedral decomposition along the path. Additionally, we study the distribution of Chebyshev centers and related radii across different polyhedra, shedding light on the polyhedral shape, size, clustering, and aiding in the understanding of decision boundaries.

## 1. Introduction

ReLU feedforward neural networks (FFNNs) exhibit a number of interesting local and global geometric properties. These networks decompose the input space into convex polyhedra and assign to each data point within the same polyhedron a common linear affine function. This polyhedral decomposition offers a fundamental geometric framework, enabling researchers to comprehend the network's partitioning and modeling of the input space. By investigating these geometric properties, including the decomposition of the input space and the counting of linear regions, researchers can gain profound insights into the expressive power, generalization abilities, and limitations of the network. The following sections will present a thorough literature review focusing on the key aspects that have been extensively examined.

The exploration of neural network mappings, which encompass diverse architectures like convolutional neural networks, residual networks, skip connected networks, and recurrent neural networks, as max-affine spline operators, has been extensively investigated by Balestriero and Baraniuk (2018). Sattelberg et al. (2020) built intuition on how the polyhedral decomposition acts and both how they can potentially be reduced in number and how similar structures occur across different neural networks. The number of polyhedra present in the input space, or within a bounded region thereof, serves as a measure of the network's expressivity and complexity. Bounds, both upper and lower, on the maximum number of attainable polyhedra for a given ReLU FFNN architecture can be found in multiple studies such as Pascanu et al. (2013), Montufar et al. (2014), Raghu et al. (2017), Arora et al. (2018), Serra et al. (2018), Hanin and Rolnick (2019), Hinz and van de Geer (2019), and Safran et al. (2022). In an alternative approach, Wang (2022) investigated local properties of the polyhedra, such as inspheres, hyperplane directions, decision boundaries, and the relevance of surrounding regions, to analyze the behavior of neural networks. Masden (2022) has given a full encoding of the canonical polyhedral complex across all dimensions.

Various algorithms in Xiang et al. (2018), Yang et al. (2020), and Xu et al. (2021) have been devised to compute the precise polyhedral decomposition of the input space by employing a layer-by-layer linear inequality solving approach. For larger decomposition instances, an efficient method proposed by Vincent and Schwager (2021) and Liu et al. (2023), is available, which systematically enumerates all polyhedra within the input space by traversing the neighbors of each polyhedron.

Several studies have delved into the intricate geometric properties of ReLU feedforward neural networks. Notably, Zhang et al. (2018) established a profound connection between ReLU FFNNs and tropical geometry, showcasing their equivalence to tropical rational maps. Ergen and Pilanci (2021) focused their attention on finite-width two-layer ReLU networks and revealed that optimal solutions to the regularized training problem can be characterized as extreme points within a convex set, capitalizing on the advantageous attributes of convex geometry. Additionally, Novak et al. (2018) conducted meticulous sensitivity analyses on trained neural networks, scrutinizing the input-output Jacobian norm and the quantification of linear regions in the realm of image classification tasks.

The research conducted by Jamil et al. (2022) and Jamil et al. (2023) has presented a binary vector representation for individual polyhedra, emphasizing its ability to capture abundant information related to both the data and the neural network. Their research has compellingly demonstrated the practical utility of these binary vectors as highly effective tools for enhancing the explainability of neural networks and facilitating the detection of adversarial instances. Building upon the foundational research mentioned earlier, our primary objective is to construct a comprehensive toolbox that effectively leverages the binary vectors and the associated linear model for polyhedra. By harnessing these tools, we aim to delve into and analyze the intricate geometric properties exhibited by ReLU FFNNs.

In this manuscript, we make several key contributions. Firstly, we formulated the codebase for the toolbox as outlined in our prior work (Liu et al., 2023). This codebase is now accessible to the public, and it can be found at the following URL: https://github.com/cglrtrgy/GoL_Toolbox. Leveraging this toolbox, we delve into the intricate geometries of neural networks, utilizing the Hamming distance as a dissimilarity metric for binary vectors to gain insights into network geometry. Additionally, we employ the bisection method to generate samples with Hamming distances of 1, revealing network connectivity. We further explore Chebyshev centers and polyhedral radii, shedding light on polyhedral shape and size, network clustering, decision boundaries, and generalization capabilities. Our approach is validated through implementations on toy datasets, MNIST, and CIFAR-10 datasets, offering compelling insights into neural network geometries.

The remaining sections of the paper are organized as follows: Section 2 introduces the toolbox, providing a comprehensive overview of its functionalities. Section 3 details the methodologies employed for analyzing the geometries of neural networks. It covers the distance metric used, the application of the bisection method, and the utilization of Chebyshev center analysis. In Section 4, 5, the toolbox and the aforementioned analysis methods are demonstrated through illustrative examples using both toy datasets, the MNIST, and CIFAR-10 dataset. Finally, Section 6 provides a conclusive summary of the paper.

## 2. Definitions and methods

In this section, we will provide a comprehensive review of the following key aspects: model of ReLU FFNNs, the definition of binary vectors, the linear model for polyhedra decomposed by ReLU FFNNs, and the traversal method employed for listing these decomposed polyhedra. For more in-depth information and references, please refer to Liu et al. (2023).

### 2.1. ReLU feedforward neural network (FFNNs)

We consider an (*L*+1)-layer FFNN with an input space denoted as ℝ^*m*^ and an output space denoted as ℝ^*n*^. Each hidden layer consists of *h*_*i*_ nodes. The weight matrix and bias vector of layer *i* are denoted as $W_{i}\in {\mathbb{R}}^{h_{i}\times h_{i-1}}$ and $b_{i}\in {\mathbb{R}}^{h_{i}}$, respectively. The ReLU activation function is applied to the initial *L* layers, while the final layer does not have an activation function. For a given input *x*∈ℝ^*m*^, the output in layer *i* is denoted as $F_{i}\left(x\right)\in {\mathbb{R}}^{h_{i}}$. The given notation represents the FFNN as follows:


(1) $$\begin{array}{l} {\mathbb{R}}^{m}\overset{\left(W_{1},b_{1}\right)}{\underset{\text{ReLU}}{\to }}{\mathbb{R}}^{h_{1}}\overset{\left(W_{2},b_{2}\right)}{\underset{\text{ReLU}}{\to }}{\mathbb{R}}^{h_{2}}\to \ldots \to \\ \;{\mathbb{R}}^{h_{L-1}}\overset{\left(W_{L},b_{L}\right)}{\underset{\text{ReLU}}{\to }}{\mathbb{R}}^{h_{L}}\overset{\left(W_{L+1},b_{L+1}\right)}{\underset{}{\to }}{\mathbb{R}}^{n}. \end{array}$$

The feedforward process of model (1) can be summarized as follows:

1). Layer 0 (Input Layer): Given a data point *x* ∈ ℝ^*m*^, it serves as the input to Layer 1.

2). Layer 1 to *L* (Hidden Layers): The output of *x* at layer *i* can be expressed as:


(2) $$\begin{array}{l} F_{i}(x)=\text{ReLU}(W_{i}F_{i-1}(x)+b_{i})\\ \;=\left[\begin{array}{c} \max\{0,w_{i,1}F_{i-1}(x)+b_{i,1}\}\\ \vdots \\ \max\{0,w_{i,h_{i}}F_{i-1}(x)+b_{i,h_{i}}\} \end{array}\right], \end{array}$$

where *w*_*i, j*_ is the *j*th row of *W*_*i*_ and *b*_*i, j*_ is the *j*th entry of *b*_*i*_.

3). Layer *L*+1 (Output Layer): The output of *x* at layer *L*+1 (also the output of the FFNN) is *W*_*L*+1_*F*_*L*_(*x*)+*b*_*L*+1_. By iteration, this implies that an FFNN represents an affine mapping given a data point *x*.

### 2.2. Binary vector

A ReLU feedforward neural network performs a partitioning of the input space into convex polyhedra (Sattelberg et al., 2020), where each individual polyhedron is associated with a corresponding binary vector representation (Liu et al., 2023). The binary vector is defined based on the output of the ReLU activation function in each hidden layer of model (1). The definition is as follows:

For a given point *x* ∈ ℝ^*m*^ to model (1), its binary vector at hidden layer *i* is defined as


$$s_{i}\left(x\right)={\left[s_{i,1}\left(x\right)\;\ldots \;s_{i,h_{i}}\left(x\right)\right]}^{⊤}\in {\mathbb{R}}^{h_{i}},$$

where *s*_*i, j*_(*x*) (with 1 ≤ *j* ≤ *h*_*i*_) is defined as follows:


(3) $$s_{i,j}(x)=\{\begin{array}{ll} 1 & \text{if }w_{i,j}F_{i-1}(x)+b_{i,j}>0,\\ 0 & \text{if }w_{i,j}F_{i-1}(x)+b_{i,j}\leq 0. \end{array}$$

The binary vector of *x* in model (1) is obtained by stacking its binary vectors from all hidden layers as follows:


(4) $$\begin{array}{l} s\left(x\right)={\left[s_{1}^{⊤}\left(x\right)\;\ldots \;s_{L}^{⊤}\left(x\right)\right]}^{⊤}\in {\mathbb{R}}^{h}, \end{array}$$

where $h=\sum_{i=1}^{L}h_{i}$ is the total number of nodes across all hidden layers.

It is worth noting that points residing within the same polyhedron exhibit identical binary vectors, thereby allowing each polyhedron to be represented by a single binary vector. Subsequently, the forthcoming section will provide a review of the linear model expressed in terms of the binary vector associated with each polyhedron.

### 2.3. Linear model for polyhedra

Given a data point *x*, we assume that its binary vector *s*(*x*) is obtained using Equation (4). To ensure consistent directionality in expressing the linear inequalities, a sign vector $s_{i}^{\prime }={\left[s_{i,1}^{\prime }\;\ldots \;s_{i,h_{i}}^{\prime }\right]}^{⊤}$ is defined for each hidden layer *i*, where $s_{i,j}^{\prime }=1$ if *s*_*i, j*_ = 0 and $s_{i,j}^{\prime }=-1$ if *s*_*i, j*_ = 1.

Let Ŵ_*j*_ = *W*_*j*_diag(*s*_*j*−1_)Ŵ_*j*−1_ and ${\hat{b}}_{j}=W_{j}\text{diag}\left(s_{j-1}\right){\hat{b}}_{j-1}+b_{j}$ for 2 ≤ *j* ≤ *L* with ${Ŵ}_{1}=W_{1},{\hat{b}}_{1}=b_{1}$. The linear model for the polyhedron associated with the binary vector *s*(*x*) is expressed as follows:


(5) $$\begin{array}{l} Ax\leq c, \end{array}$$

where $A={\left[A_{1}^{⊤}\;A_{2}^{⊤}\ldots \;A_{L}^{⊤}\right]}^{⊤}\;\text{and}\;c={\left[c_{1}^{⊤}\;c_{2}^{⊤}\ldots \;c_{L}^{⊤}\right]}^{⊤}$ with $A_{j}=\text{diag}\left(s_{j}^{\prime }\right){Ŵ}_{j}\in {\mathbb{R}}^{h_{i}\times m}$ and $c_{j}=\text{diag}\left(s_{j}^{\prime }\right)\left(-{\hat{b}}_{j}\right)\in {\mathbb{R}}^{h_{i}}$.

It's essential to highlight that, within the polyhedron defined by the bit vector *s*, the output of any input *x* is solely determined by a single affine mapping: $G\left(x\right)=W_{L+1}\text{diag}\left(s_{L}\right){Ŵ}_{L}x+W_{L+1}\text{diag}\left(s_{L}\right){\hat{b}}_{L}+b_{L+1}$.

Each facet of the polyhedron corresponds to a unique linear inequality from (5), indicating the non-redundancy of these inequalities. An active bit within the *i*th entry of *s*(*x*) indicates that the *i*th linear inequality is essential and not redundant. The following linear program can be used to determine whether the *i*th linear inequality of (5) is redundant or not.

Let


$$A={\left[\begin{array}{c} a_{1}\;a_{2}\;\ldots \;a_{h} \end{array}\right]}^{⊤}\;\text{and }c={\left[\begin{array}{c} c^{1}\;c^{2}\;\ldots \;c^{h} \end{array}\right]}^{⊤}$$

with $a_{i}\in {\mathbb{R}}^{m}$ and *c*^*i*^∈ℝ. We define Ã as the matrix obtained by removing the *i*th row $a_{i}^{⊤}$ from *A*, and $\tilde{c}$ as the vector obtained by removing the *i*th element *c*^*i*^ from *c*. Consider the following linear program


(6) $$\begin{array}{l} \underset{x}{\text{maximize}}\;a_{i}^{⊤}x\\ \text{s}.\text{ t}.\;\tilde{A}x\leq \tilde{c}. \end{array}$$

If the optimal objective value of (6) is less than or equal to *c*^*i*^, it indicates that the *i*th linear inequality is redundant. In such cases, we can remove the row $a_{i}^{⊤}$ and the corresponding element *c*^*i*^ from *A* and *c*, respectively. This iterative process of removing redundant constraints leads to the formation of the reduced set (*A*′, *c*′), which represents the minimum set of constraints in (*A, c*). Moreover, the indices of the active bits in *s*(*x*) can be obtained through this process. It is noteworthy that the number of active bits within a binary vector corresponds to the number of nonredundant inequalities present in its linear model (5).

By flipping an active bit in *s*(*x*) (switching 1 to 0 or 0 to 1), a binary vector corresponding to a neighboring polyhedron that shares a facet with the given polyhedron can be obtained. The validity of this claim can be demonstrated through a proof by contradiction. This enables the derivation of the binary vectors that determine all polyhedra decomposed by the neural network, along with the corresponding derivation of the associated linear models. This method, known as the traversal method, will be reviewed in the subsequent section.

### 2.4. Traversal-and-Pruning method

In this section, we shall present the Traversal-and-Pruning method as outlined in Algorithm 1. This method systematically explores all bit vectors that define a polyhedron within the input space through the activation of specific bits.

**Algorithm 1:**
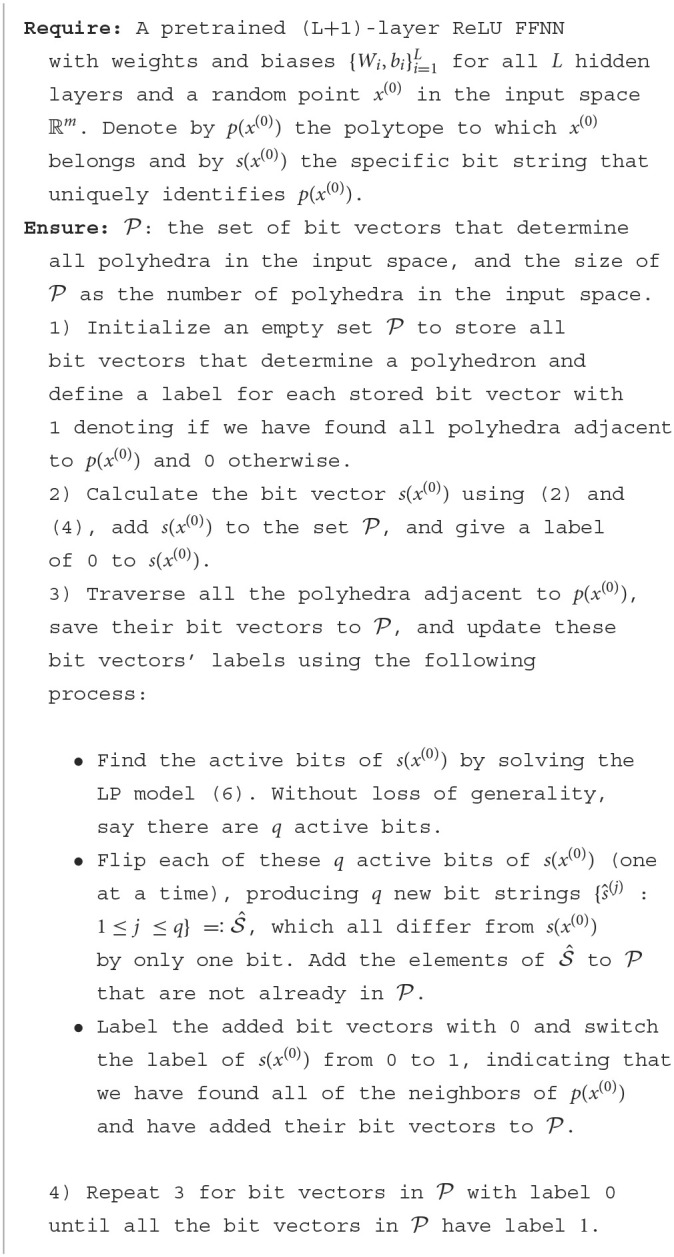
Traversal-and-Pruning method.

The method employed in this approach originates from Liu et al. (2023), while the concept of flipping an active bit aligns with the findings presented in Vincent and Schwager (2021). However, the key distinction lies in the fact that this method generates binary vectors for all polyhedra, which holds significant importance in the subsequent section as it facilitates the exploration of the neural network's underlying geometry. Furthermore, Liu et al. (2023) also demonstrates the capability to enumerate polyhedra within a bounded region, although specific details are omitted in this context.

The traversal method exhaustively enumerates all binary vectors, and this assertion can be substantiated through the following argument: Consider a set of binary vectors represented as vertices in a graph, where two vertices are connected if they differ by flipping a single active bit.

- We begin by selecting an initial binary vector arbitrarily. The method identifies the active bits in this vector, flips one active bit at a time to generate neighbors, and continues this process iteratively until all possible neighbors are explored.
- To prove completeness, we employ mathematical induction. In the base case, we establish that the method successfully traverses all binary vectors within a small neighborhood of the initial vector. By the inductive hypothesis, we assume that for any binary vector within a certain radius of the initial vector, the traversal method can reach it through a sequence of active bit flips.
- For the inductive step, we show that the method can extend this reach to binary vectors within an expanded radius. By flipping active bits, we demonstrate that it's possible to reach any binary vector within this extended region. This ensures that the method systematically explores the entire space of binary vectors.
- Moreover, the graph formed by these binary vectors is connected because any binary vector in the graph can be transformed into any other binary vector by a series of single-bit flips, ensuring the existence of a path between any two binary vectors.

As a result, we conclude that the traversal method, starting from an arbitrary binary vector, effectively enumerates all binary vectors in the defined space by flipping active bits. This proof establishes the method's capability to traverse and enumerate all binary vectors systematically.

## 3. Geometric aspects and methodologies of neural networks

In this section, utilizing the toolbox developed in Section 2, our primary aim is to thoroughly investigate the intricate geometries that underlie neural networks. To achieve this, we leverage the Hamming distance, which serves as a dissimilarity metric based on the binary vectors. Moreover, we employ the bisection method to identify the sample points along the shortest Euclidean path between two given data points, imposing the constraint that neighboring sample points must exhibit a Hamming distance of 1. Additionally, we explore the Chebyshev centers and the corresponding radii of the polyhedra, providing valuable insights into the characteristics of the polyhedral structures.

### 3.1. Distance metric

The Euclidean distance or *L*_2_ norm is widely adopted as the primary distance metric between two data points, including images. Alternatively, for binary data, the Hamming distance is commonly employed, quantifying the dissimilarity between two binary strings by counting the differing positions. In this manuscript, we establish the Hamming distance between two data points or polyhedra as the count of dissimilar entries in their respective binary vectors, which are obtained using Equation (4) based on a pre-trained FFNN.

It is important to highlight that the Hamming distance between polyhedra serves as an approximation for quantifying the minimum number of steps required to transition between two polyhedra. This observation establishes the Hamming distance as a valuable metric for capturing the geometric relationship and connectivity among polyhedra. Notably, the effectiveness of the Hamming distance in unveiling the underlying mechanisms of neural network functionality has been substantiated in Jamil et al. (2023). Inspired by these findings, we leverage the Hamming distance between data points or polyhedra to probe the geometric characteristics of a pretrained FFNN.

### 3.2. Bisection method

The Hamming distance serves as an estimate for determining the shortest path between vertices on the dual graph of the polyhedral decomposition. In our case, we focus on finding the samples along the shortest Euclidean path given two data points, ensuring that the Hamming distance between adjacent samples is precisely 1. To achieve this, we introduce a bisection method, described in Algorithm 2, that allows us to generate data points and their corresponding binary vectors between any two given data points, while satisfying the following properties:

**Algorithm 2:**
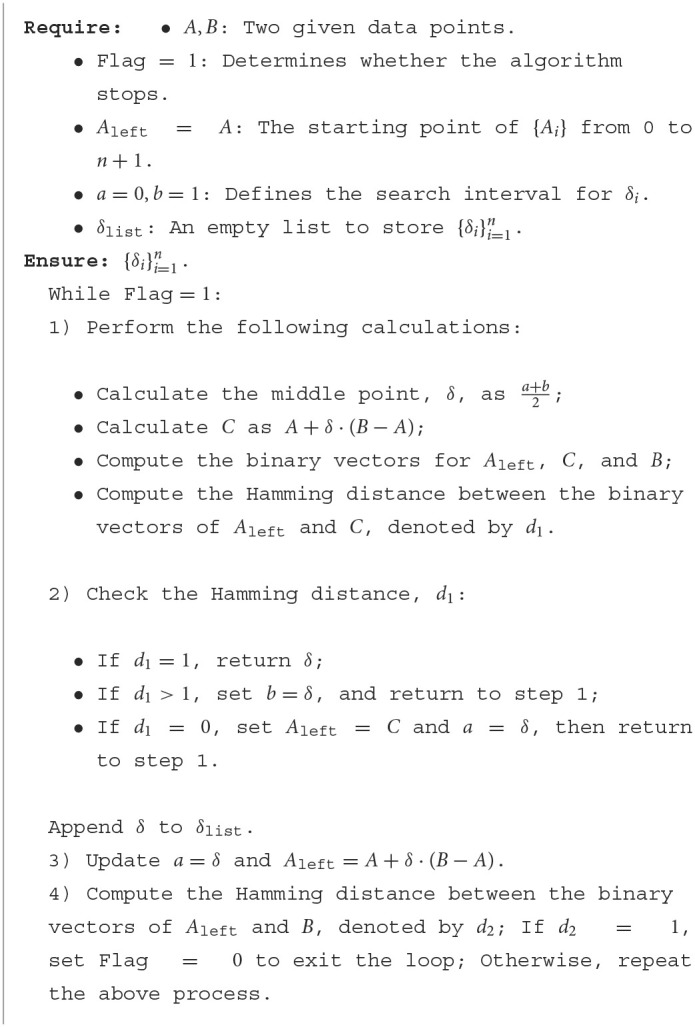
Bisection method.

(1) The Hamming distance between two adjacent data points is exactly 1.

(2) The sampled data points align with the same line defined by the initial two data points.

Formulating this mathematically, we consider two data points, denoted as *A* and *B*, within the input space. Our objective is to identify a series of points ${\left\{A_{i}\right\}}_{i=1}^{n}$ that meet the following criteria:

(1) *A*_*i*_ = *A*+δ_i_*(*B*−*A*), where δ_*i*_∈(0, 1) for 1 ≤ *i* ≤ *n*. Here, we set *A*_0_ = *A* and *A*_*n*+1_ = *B*.

(2) The Hamming distance between the bit vectors of *A*_*i*_ and *A*_*i*−1_, for 1 ≤ *i* ≤ *n*+1, is equal to 1.

### 3.3. Chebyshev center

In our exploration of convex polyhedra in ℝ^*N*^, various statistical characteristics are of interest. For example, determining the number of *d*-dimensional faces in *P* or calculating its volume is a fundamental pursuit. The count of (*N*−1)-dimensional faces, analogous to the number of active bits, is computable via linear programming. On the contrary, pinpointing the number of zero-dimensional faces (i.e., vertices) poses challenges due to combinatorial complexities. To illustrate, a convex polytope with 30 faces in ℝ^15^ can possess over 150,000 vertices, rendering calculations intractable in ℝ^1000^. Estimating the radius and center of the largest inscribed sphere (the Chebyshev center) is achievable through linear programming, while determining the radius and center of the smallest bounding sphere remains infeasible. Similarly, exact volume calculations elude us, but the Chebyshev center provides a coarse approximation. The Gaussian mean width offers another proxy for volume but relies on probabilistic algorithms. Leveraging the Chebyshev center as a representation of the polyhedron's “center” and the associated sphere's radius as a volume indicator, we gain insights into polyhedral attributes, network clustering, decision boundaries, and generalization capabilities.

Consider a bounded set *Q*. The Chebyshev center refers to the center of the largest inscribed ball within *Q*, as defined in Boyd and Vandenberghe (2004). In our case, we aim to determine the Chebyshev center and its corresponding radius for a polyhedron that has been decomposed by a pretrained FFNN.

Assume that *A*′*x* ≤ *c*′ represents a minimal set defining a bounded polyhedron resulting from the decomposition performed by an FFNN. Here,


$$A^{\prime }={\left[\begin{array}{c} a_{1}^{\prime }\;a_{2}^{\prime }\;\ldots \;a_{l}^{\prime } \end{array}\right]}^{⊤}\;\in {\mathbb{R}}^{l\times m}\;\text{and }c^{\prime }={\left[\begin{array}{c} c_{1}^{\prime }\;c_{2}^{\prime }\;\ldots \;c_{l}^{\prime } \end{array}\right]}^{⊤}\in {\mathbb{R}}^{l}.$$

Note: In the case of an unbounded polyhedron, the inclusion of bounds on each dimension can be implemented to render it bounded.

To describe the center of the ball inscribed within the polyhedron *A*′*x* ≤ *c*′, we introduce $x_{c}\in {\mathbb{R}}^{m}$ and *r*∈ℝ, where *x*_*c*_ represents the center and *r* denotes the radius. Any point within the ball can be expressed as *x*_*c*_+*t*, with ||*t*||_2_ ≤ *r*, and it must satisfy the constraints:


$${a^{\prime }}_{i}^{⊤}\left(x_{c}+t\right)\leq c_{i}^{\prime }\;\text{for }1\leq i\leq l.$$

We know that ${\text{sup}}_{\|t{\|}_{2}\leq r}\{{a^{\prime }}_{i}^{⊤}t\}=r||{a^{\prime }}_{i}|{|}_{2}$ for 1 ≤ *i* ≤ *l*, which allows us to rewrite the constraints as:


$${a^{\prime }}_{i}^{⊤}x_{c}+r||{a^{\prime }}_{i}|{|}_{2}\leq c_{i}^{\prime }\;\text{for }1\leq i\leq l.$$

Therefore, the problem of maximizing the radius *r* can be formulated as the following optimization problem:


(7) $$\begin{array}{l} \underset{r,x_{c}}{\text{maximize}}\;r\\ \text{s}.\text{ t}.\;a{'}_{i}^{⊤}x_{c}\text{+}r\text{||}a{'}_{i}{\text{||}}_{\text{2}}\leq c_{i}^{'}\text{ for 1}\leq i\leq l. \end{array}$$

Define


$$\begin{array}{l} x=\left[\begin{array}{c} r\\ x_{c} \end{array}\right]\in {\mathbb{R}}^{m+1},\;e=\left[\begin{array}{c} 1\\ 0\\ \vdots \\ 0 \end{array}\right]\in {\mathbb{R}}^{m+1},\text{ and }\hat{A}=\left[\begin{array}{cc} ||{a^{\prime }}_{1}|{|}_{2} & {a^{\prime }}_{1}^{⊤}\\ ||{a^{\prime }}_{2}|{|}_{2} & {a^{\prime }}_{1}^{⊤}\\ & \vdots \\ ||{a^{\prime }}_{l}|{|}_{2} & {a^{\prime }}_{l}^{⊤} \end{array}\right]\\ \;\in {\mathbb{R}}^{l\times (m+1)}. \end{array}$$

The optimization problem (7) can then be reformulated as:


(8) $$\begin{array}{l} \underset{x}{\text{minimize}}\;-e^{⊤}x\\ \text{s}.\text{ t}.\;\tilde{A}x\leq c'. \end{array}$$

Problem (8) is a linear program, which can be effectively solved using the cvxpy package in Python.

## 4. Examples

In this section, we initially showcase the efficacy of our toolbox using toy datasets and the MNIST dataset. Subsequently, we apply the bisection method and Chebyshev center analysis to the MNIST dataset, enabling a detailed investigation of the intricate geometries present in neural networks.

### 4.1. Basic FFNN

#### 4.1.1. Toy examples 1: 20 nodes

We initially employed model (1) composed of two hidden layers, each consisting of 10 nodes, to approximate the function $f_{1}\left(x_{1},x_{2}\right)=x_{1}^{2}+x_{2}^{2}-0.4$. The training of this model was performed using PyTorch, a Python library known for its capabilities in deep learning (Paszke et al., 2019). To create a representative dataset, we uniformly sampled 10,000 points from the interval [−1, 1]^2^. The training process iterated until a predefined early stopping criterion, based on the convergence of the validation loss, was satisfied.

**Figure 1:**
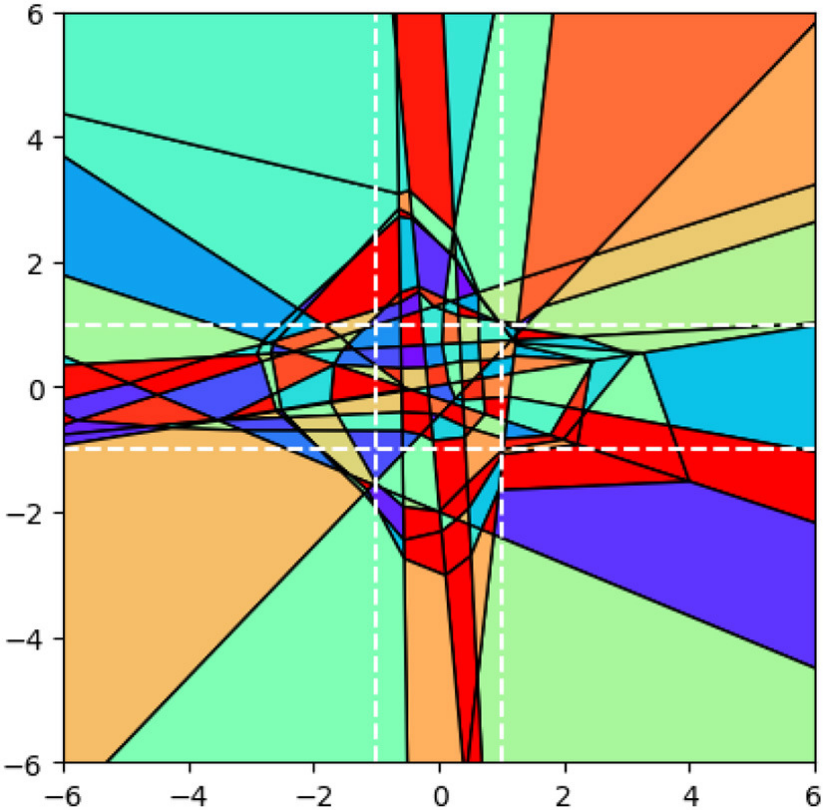
2-D visualization of polyhedral composition within [−6, 6]^2^. The central bounded region, indicated by white dotted lines, encompasses [−1, 1]^2^.

Figure 1 is generated through the following procedure: (1) Enumerate all the binary vectors using Algorithm 1. (2) Determine the linear model associated with each polyhedron using equation (5). (3) Compute the vertices of each polyhedron using the Python package intvalpy and plot each polyhedron using the vertices.

Figure 1 illustrates the decomposition of polyhedra achieved by the aforementioned model. Within the bounded region [−1, 1]^2^, Algorithm 1 yields a total of 218 polyhedra, contributing to the overall count of 237 polyhedra in the 2-dimensional space ℝ^2^. Among these polyhedra, 211 are classified as bounded, while 26 are deemed unbounded.

**Observations:** The size of the polyhedra within the training area is relatively small, and their size increases as they move farther away from the training area. Furthermore, for points located on the two white dotted lines at a fixed Euclidean distance, their Hamming distance is greater within the training area and decreases as the points move away from it. To illustrate this, consider the following example: the Hamming distance between the points (0, −1) and (0, 1) is greater than the Hamming distance between the points (6, −1) and (6, 1). This trend highlights how the Hamming distance varies with respect to the proximity to the training area.

#### 4.1.2. Toy examples 2: different model structures

The relationship between the number of polyhedra decomposed by an FFNN and the network depth/width has been extensively explored in prior studies such as Pascanu et al. (2013), Montufar et al. (2014), Raghu et al. (2017), and Hanin and Rolnick (2019). This study aims to investigate the relationship between the mean square error (MSE) of the objective function on the validation dataset and the number of polyhedra. The examination involves exploring different network structures while keeping a consistent number of nodes or layers. By analyzing this relationship, we aim to gain insights into the influence of network structure on the performance of the model.

We conducted a series of experiments where we varied the number of hidden layers while maintaining a consistent number of 5 nodes per layer. Additionally, we adjusted the number of nodes in each hidden layer while keeping a consistent total number of 3 layers. To assess the stability and consistency of the results, we repeated each scenario 50 times with different initializations. The experimental setup aligns with the details described in Section 4.1.1. The results on the polyhedra count and MSE statistics are listed in Table 1.

**Table 1:** Comparison of model architectures: polyhedra count and MSE statistics on validation dataset.

**Models**		**# Polyhedra stats**					**Model training stats**	
**# layer**	**# nodes**	**Avg**	**SD**	**Median**	**Min**	**Max**	**Avg MSE**	**SD MSE**
2	5	55.10	8.28	56	37	70	0.001389	0.000618
4	5	163.33	43.99	156.5	101	277	0.001086	0.001553
6	5	260.70	108.29	249	114	664	0.000947	0.001926
8	5	472.53	232.09	392	265	1,081	0.000593	0.000518
10	5	588.80	181.59	539.5	378	1,072	0.000635	0.000711
3	5	110.20	27.35	105	75	210	0.000651	0.000300
3	10	392.20	64.65	379	302	532	0.000081	0.000021
3	15	788.83	138.14	774	572	1,051	0.000036	0.000014
3	20	1,405.30	233.28	1,376	970	1,938	0.000019	0.000003
3	25	2,225.60	315.68	2,245.5	1,592	3,153	0.000014	0.000002

The results presented in Table 1 reveal significant trends. Firstly, when the number of nodes is held constant, increasing the number of layers leads to improved average performance and an increase in the number of polyhedra. Moreover, maintaining a fixed number of layers while increasing the number of nodes also results in improvements in both the number of polyhedra and performance.

#### 4.1.3. Toy examples 3: visualizing polyhedral compositions in 3D

**Figure 2:**
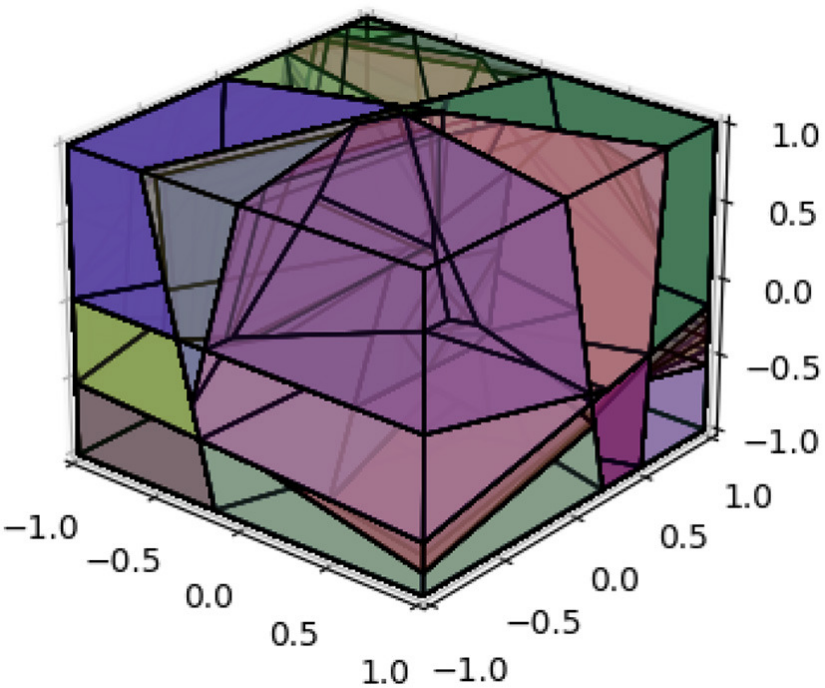
3-D visualization of polyhedral composition within [−1, 1]^3^.

Following the same procedure outlined in Section 4.1.1, we utilized a neural network model comprising three hidden layers, each containing 10 nodes, to approximate the function $f_{1}\left(x_{1},x_{2},x_{3}\right)=x_{1}^{2}+x_{2}^{2}+x_{3}^{2}-3$. To ensure an accurate representation of the function, we randomly sampled 125,000 points from the range [−1, 1]^3^. Figure 2 illustrates the decomposition of polyhedra in three-dimensional space. This decomposition encompasses a total of 2,212 polyhedra within the range of [−1, 1]^3^ in all three dimensions.

### 4.2. MNIST

In this experimental study, we conducted our analysis using the well-known MNIST dataset with dimensions of 28 × 28. Model (1), comprising five hidden layers, was trained on the training dataset using five distinct configurations. Notably, the loss function utilized was cross-entropy, optimization was conducted with the Adam optimizer, and the maximum training epochs were limited to 50. The node configurations for these layers were chosen as 300, 350, 400, 450, and 500, respectively, for each of the five structures, surpassing the input dimension of 784. Remarkably, all configurations consistently yielded training and test accuracies surpassing 98%. Subsequently, we randomly selected 30 images from the training dataset and computed their corresponding binary vectors and the linear model (5) representing the polyhedra they belong to for the five different structures. To determine the active bits for each binary vector across the five different model structures, we solved a varying number of instances of model (7), specifically 1,500, 1,750, 2,000, 2,250, and 2,500, by considering different values of *i*. The coefficient matrix Ã in the constraint of model (6) had dimensions of 1, 499 × 784, 1, 749 × 784, 1, 999 × 784, 2, 249 × 784, and 2, 499 × 784 for the respective instances. To optimize computational efficiency, we leveraged parallel processing on a Linux machine equipped with AMD EPYC 7,452 2.35 GHz processors, utilizing 48 CPUs to efficiently solve model (6) for the corresponding number of times: 1,500, 1,750, 2,000, 2,250, and 2,500, respectively.

Table 2 provides a comprehensive summary of the experimental results, presenting various metrics for different configurations represented by the “Variable nodes per hidden layer” columns. The table includes measurements such as the average (Avg), standard deviation (SD), minimum (Min), and maximum (Max) number of active bits.

**Table 2:** Results of active bits for different model structures.

	**Variable nodes per hidden layer**				
	**300 nodes**	**350 nodes**	**400 nodes**	**450 nodes**	**500 nodes**
Avg # active bits	658.30	740.57	780.00	886.87	975.00
SD # active bits	64.07	82.18	97.48	105.19	124.29
Min # active bits	520	525	556	636	719
Max # active bits	785	885	942	1,056	1,181

The results summarized in Table 2 reveal the following insights: As the number of nodes per hidden layer increases (from 300 to 500), the average number of active bits also increases, indicating a positive correlation between the number of nodes and the number of polyhedra; The standard deviation of active bits shows some variation across different configurations, with larger numbers of nodes generally leading to higher variability. The minimum and maximum number of active bits demonstrate an increasing trend as the number of nodes per hidden layer increases.

**Figure 3:**
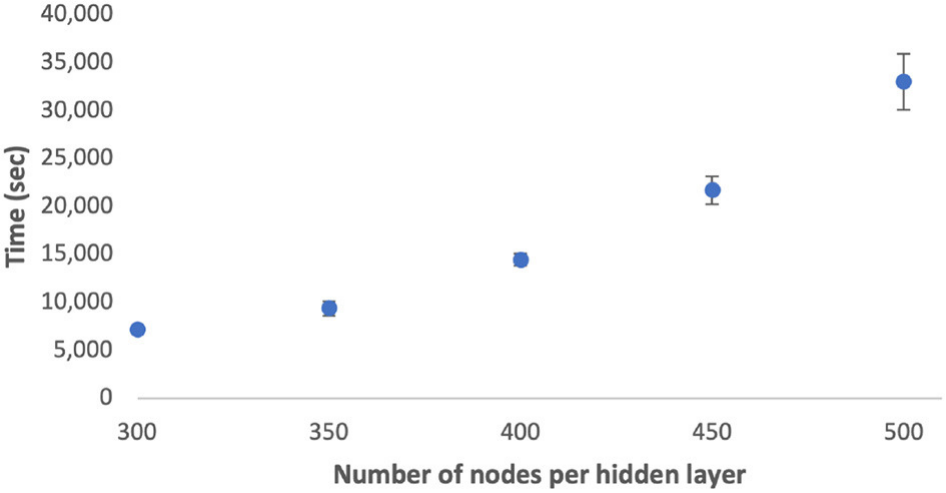
Time taken to find active bits vs. node sizes per hidden layer. Error bars represent 1 SD.

The computational complexity for solving problem (6) can be described as $O\left(m^{3}h\right)$. Figure 3 showcases the average and standard deviation of computation time across various node quantities using the Python cvxpy solver. The results demonstrate a positive correlation between the number of nodes per hidden layer and the average computation time. Furthermore, configurations with a larger number of nodes demonstrate increased variability in computation time. In future work, we plan to investigate the scalability of these computations on GPUs.

#### 4.2.1. Hamming distance and bisection method

In this section, we begin by showcasing the efficacy of the Hamming distance in capturing data features and its properties across different source points. We then utilize the bisection method to generate samples along the shortest Euclidean distance path between two designated data points, subsequently analyzing the fluctuations in the number of polyhedra among nearest neighbors as the neural network's layer count undergoes variation.

##### 4.2.1.1. Why using hamming distance?

In this section, we aim to elucidate the rationale behind utilizing the Hamming distance as a representation for the neural network, subsequently leveraging it to delve into the intricate geometric properties inherent within the network.

We examine three distinct structures of the neural network (1). The first comprises 3 layers with 200 nodes per layer, the second consists of 5 layers with 300 nodes per layer, and the third is composed of 5 layers with 500 nodes per layer. The training accuracies for the three structures are 99.08%, 99.01%, and 99.11%, respectively. Correspondingly, the test accuracies for these structures are 97.57%, 97.95%, and 97.64%. We compute the binary vectors corresponding to each training and test data point for the three structures. Subsequently, we determine the nearest neighbor for each data point in both the training and test sets, employing both Euclidean and Hamming distances. Additionally, we investigate whether the nearest neighbor belongs to the same class as the data point under consideration. Table 3 presents the classification accuracy rates of the nearest neighbors for training and test data points belonging to the same class, using both Euclidean distance and Hamming distance for the three structures.

**Table 3:** Classification accuracy rates of the nearest neighbors for training/test data using Euclidean and Hamming distances.

	**Data points**	
**Metrics**	**Training**	**Test**
**Euclidean**	**97.37%**	**95.58%**
Hamming for structure 1	99.50%	98.26%
Hamming for structure 2	99.52%	98.06%
Hamming for structure 3	99.58%	98.05%

From the table, we observe that the Hamming distance yields higher accuracy rates compared to the Euclidean distance for both training and test data. This suggests that the utilization of the Hamming distance measure leads to more precise classification of data points, resulting in superior classification accuracy rates compared to those obtained with the Euclidean distance measure. The superior performance of the Hamming distance can be attributed to its calculation based on binary vectors, which are derived from the pretrained neural networks. By considering only the differing entries between binary vectors, the Hamming distance captures crucial features that are highly relevant for determining similarity within the dataset. Consequently, it effectively discriminates between data points and contributes to the improved classification accuracy observed in the results.

Figure 1 illustrates a notable observation: data points that maintain a constant Euclidean distance can exhibit varying Hamming distances as they traverse the input space. This intriguing finding motivates us to explore the potential differences in the Hamming distance when comparing a data point to a sampled data point, while maintaining a fixed Euclidean distance. Specifically, we aim to explore whether the Hamming distance varies based on whether the data point is sourced from the training, test, or adversarial set.

We compute the Hamming distance between a given data point and a sampled data point, while maintaining a fixed Euclidean distance of 0.5, 0.75, and 1, respectively. This analysis encompasses data points sourced from the training, test, and adversarial datasets. The adversarial dataset is generated using the Fast Gradient Sign Method (FGSM) (Goodfellow et al., 2015) and is derived from the test dataset. The results presented herein are based on a dataset comprising 10,000 data points from the training set, as well as the entire test and corresponding adversarial datasets.

Table 4 demonstrates that the average Hamming distance between training data points and their sampled counterparts mirrors that of test data points and their corresponding samples. This consistent behavior underscores the Hamming distance's efficacy in capturing fundamental features and affinities across data points, regardless of their belonging to the training or test set.

**Table 4:** Comparison of Hamming distance between data points from training/test/adversarial datasets and sampled data points with fixed Euclidean distance.

	**Euclidean distance**					
	**0.5**		**0.75**		**1.0**	
	**Hamming distance**		**Hamming distance**		**Hamming distance**	
**Datasets**	**Avg**	**SD**	**Avg**	**SD**	**Avg**	**SD**
Training (Structure 1)	52.97	13.80	102.76	30.90	131.82	35.73
Test (Structure 1)	52.48	13.66	101.40	29.71	130.51	34.43
FGSM (Structure 1)	75.27	13.37	128.01	35.27	151.09	36.89
Training (Structure 2)	99.97	41.88	173.59	69.61	251.73	93.69
Test (Structure 2)	99.36	42.65	170.45	67.51	246.99	90.01
FGSM (Structure 2)	178.57	83.69	264.66	99.79	339.07	106.60
Training (Structure 3)	129.98	56.91	234.34	92.26	329.89	107.36
Test (Structure 3)	129.05	56.73	242.05	87.36	344.97	106.26
FGSM (Structure 3)	219.92	99.16	351.10	113.61	439.77	118.09

However, a notable disparity emerges in the Hamming distances between adversarial data points and their corresponding samples, compared to those of training and test data points. This discrepancy suggests a distinctive and divergent relationship in terms of their binary vector representations. These observations highlight that adversarial examples manifest a substantially dissimilar geometric nature compared to the original training and test data points.

The larger Hamming distances between adversarial data points and their sampled counterparts signify heightened dissimilarity and divergence in their binary vectors. This underscores that adversarial data points occupy a distinct region in the input space separate from both training and test data.

Despite the average Hamming distance between training/test data points and their samples being smaller than that between adversarial data points and corresponding samples, an overlap within the range exists. Consequently, the Hamming distance alone cannot definitively discern the adversarial nature of a point.

##### 4.2.1.2. Bisection method

In this section, we used the bisection method (Section 3.2) to systematically enumerate samples along the shortest Euclidean path between two specified data points. Initially, we analyzed the correlation between the Hamming distance and the number of polyhedra along this path, considering a given data point and its nearest neighbors in the training dataset. Furthermore, we explored how the number of polyhedra changes along the shortest Euclidean path between two nearest neighbors from the training dataset with increasing layer numbers.

Firstly, we aim to demonstrate that the Hamming distance of two given data points does not equate to the number of polyhedra along the shortest Euclidean path between these two given data points. To accomplish this, we computed the Hamming distance for six pairs of nearest neighbors within the train dataset and the results are listed in Table 5. The experiment was carried out using the pretrained model Structure 2, as discussed in the preceding section.

**Table 5:** The Hamming distance vs. the number of polyhedra along shortest Euclidean path length.

**Pairs number**	**1**	**2**	**3**	**4**	**5**	**6**
Hamming distance	98	132	184	232	282	320
# of polyhedra along the shortest Euclidean path	100	135	193	252	299	351

Table 5 illustrates the relationship between the Hamming distance and the number of polyhedra along the shortest Euclidean path between two nearest neighbors (in terms of the Hamming distance). It reveals that when the Hamming distance is small, there is a close correspondence between the Hamming distance and the number of polyhedra along the shortest Euclidean path. However, as the Hamming distance increases, the disparity between the Hamming distance and the number of polyhedra along the shortest Euclidean path becomes more pronounced.

Next, we apply the bisection method to 1000 pairs of nearest neighbors from the training dataset to investigate the variation in the number of polyhedra along the shortest Euclidean distance as the number of layers increases. For this analysis, we utilize the Euclidean distance to determine nearest neighbors, as it remains consistent regardless of any alteration in the neural network structure. We increase the number of layers from 1 to 5 and keep the number of nodes in each layer as 200 for the neural network (1).

**Figure 4:**
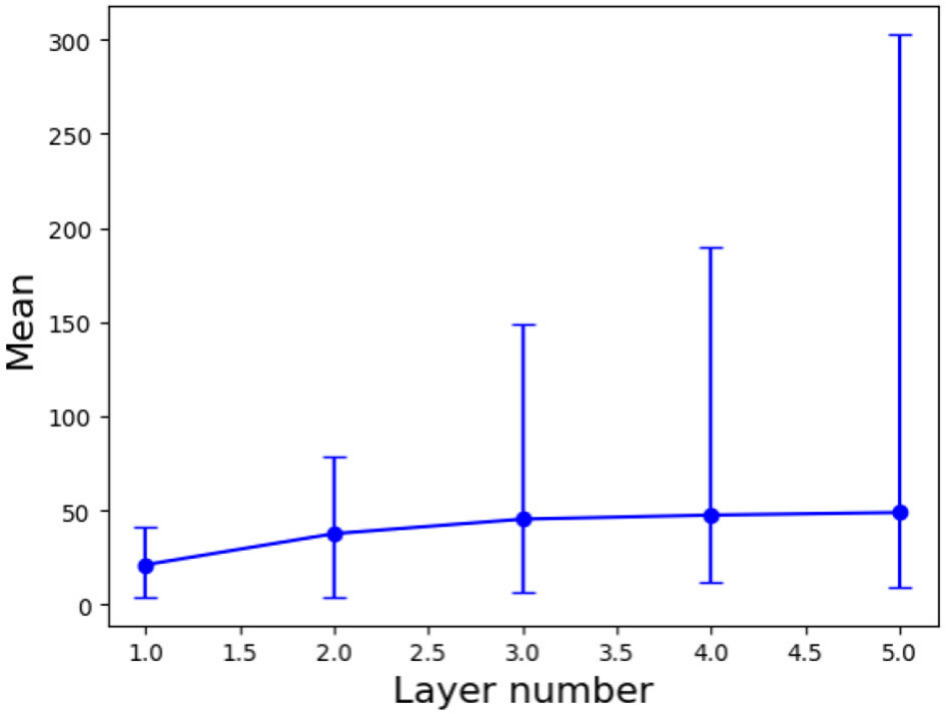
Mean values with error bars (maximum and minimum) for the number of polyhedra along the shortest Euclidean path for 1000 pairs of two nearest neighbors versus the number of layers.

Figure 4 illustrates the relationship between the number of layers and the number of polyhedra along the shortest Euclidean path between two nearest neighbors. The results reveal an exponential increase in the maximum number of polyhedra, ranging from 41 to 303, as the number of layers is increased. In contrast, the mean number of polyhedra shows a gradual rise from 21 to 49. This discrepancy suggests that for nearest neighbor pairs with larger Euclidean distances, the number of polyhedra changes significantly with the addition of layers, while most nearest neighbor pairs exhibit a relatively slow change in the number of polyhedra as the number of layers increases.

#### 4.2.2. Chebyshev center

**Figure 5:**
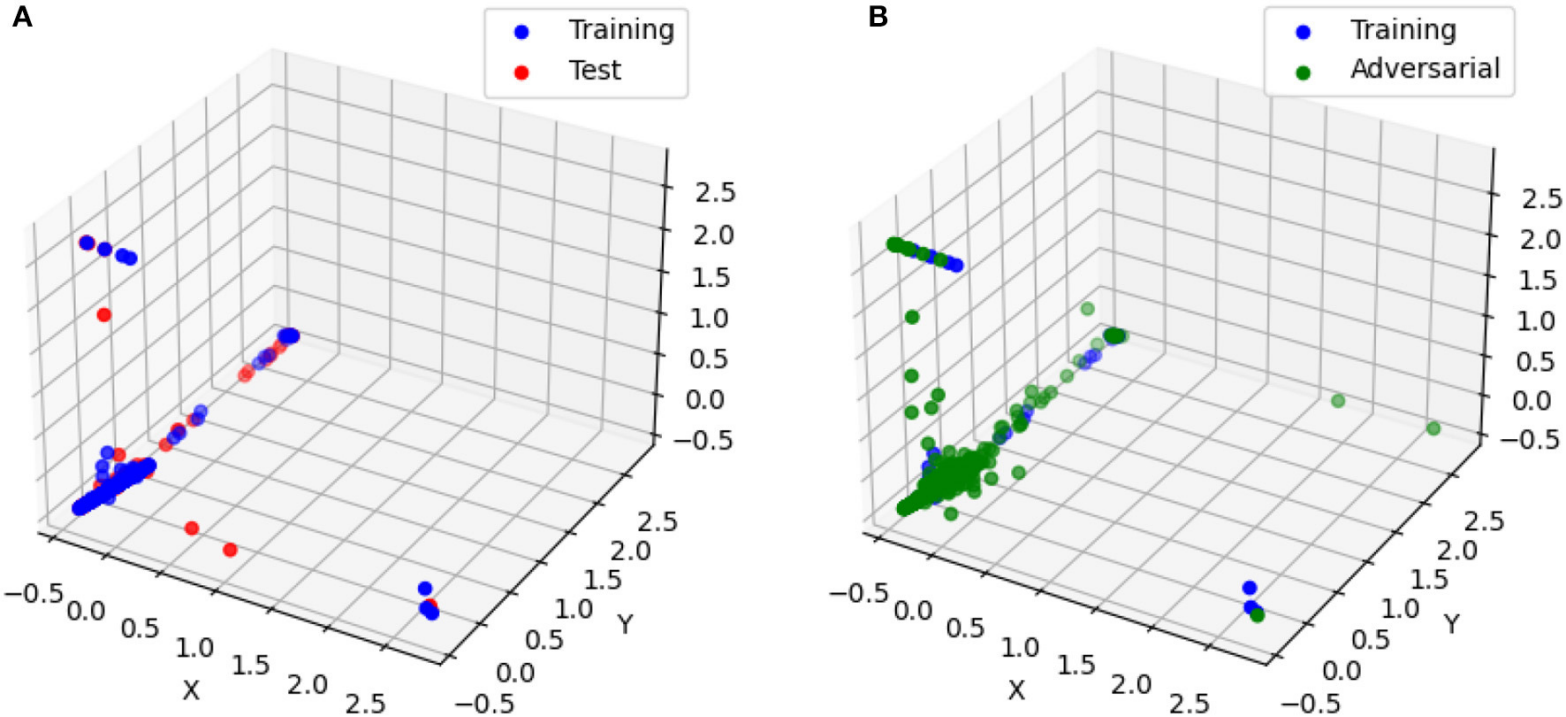
The Chebyshev center for randomly selected training, test, and adversarial samples. **(A)** The Chebyshev center for training and test samples. **(B)** The Chebyshev center for training and adversarial samples.

**Figure 6:**
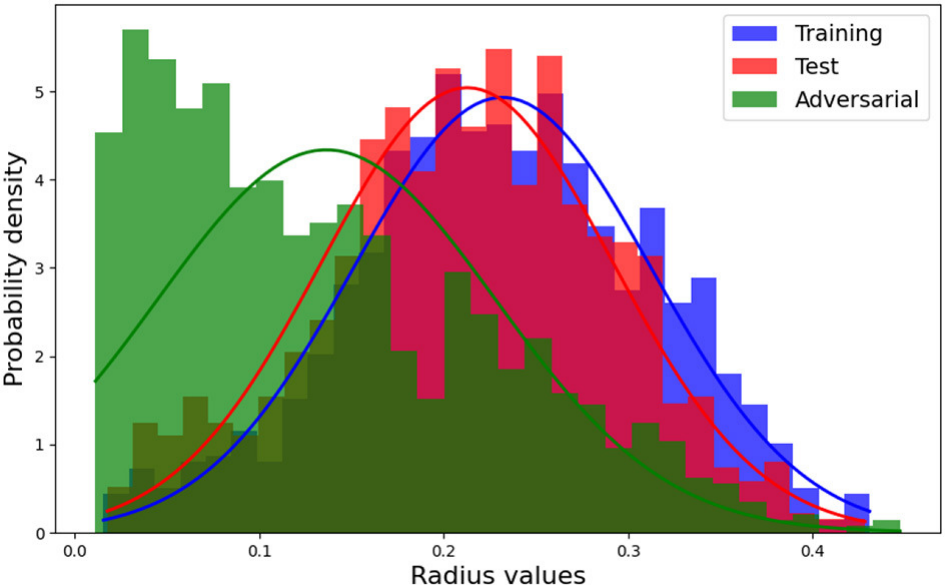
Histogram with Gaussian normal distribution for the radius of training, test, and adversarial datasets.

In this section, we employed the same pretrained models discussed in Section 4.2.1.1, along with the MNIST dataset, to conduct our analysis. Specifically, we randomly sampled 1000 data points from the training, test, and adversarial datasets. For each of these data points, we computed the linear models (6) corresponding to the polyhedra on which they reside. Additionally, we utilized model (8) to solve for the Chebyshev centers and their associated radii. The corresponding results for Structure 2 are presented in Figure 5 and Table 6. Additionally, Figure 6 showcases the distribution of radii for the 1000 data points across the three datasets. It is worth noting that the Chebyshev center for each polyhedron resides in a 784-dimensional space, while Figure 5 provides a visual representation limited to three dimensions.

**Table 6:** Statistics of the radius of the largest inscribed ball within polyhedra: training, test, and adversarial datasets.

	**Mean**	**SD**	**Max**	**Min**
Train	0.23	0.081	0.43	0.016
Test	0.21	0.079	0.43	0.018
Adv	0.13	0.092	0.54	0.097

Figure 5A visually depicts the close proximity of the Chebyshev center between the polyhedra containing randomly selected training and test samples. Additionally, Table 6 and Figure 6 present the similarity in size between the polyhedra for the training and test samples. Conversely, Figure 5B reveals a noticeable disparity in the Chebyshev center between the polyhedra encompassing the randomly selected training and adversarial data points. Furthermore, Table 6 and Figure 6 highlight the comparatively smaller size of the polyhedra housing the adversarial data samples in relation to the polyhedra encompassing the training and test data samples. These findings are consistent with the observations from Table 4, which indicates larger Hamming distances when sampling the original points from the adversarial dataset, while maintaining a fixed Euclidean distance.

The above observations provide insights into the behavior and characteristics of the neural network.

The close proximity of the Chebyshev centers and the similarity in size between the polyhedra containing the training and test samples suggest that the neural network exhibits consistent behavior and decision boundaries for these two datasets. This indicates that the network generalizes well and maintains stability in its predictions when presented with new test samples.

On the other hand, the noticeable disparity in the Chebyshev centers and the smaller size of the polyhedra for the adversarial data points indicate that the neural network behaves differently when faced with adversarial inputs. Adversarial examples are intentionally designed to mislead the network and exploit vulnerabilities in its decision-making process. The observed differences in the Chebyshev centers and polyhedra sizes suggest that the network's decision boundaries are more susceptible to manipulation and exhibit variations in response to adversarial inputs.

## 5. CIFAR-10

To demonstrate the practical applicability of our toolbox and the methodologies outlined in Sections 2 and 3, we conducted experiments using the CIFAR-10 dataset. CIFAR-10 is characterized by its inclusion of real-world images that exhibit heightened complexity, encompassing variations in lighting, orientation, and backgrounds, a notable departure from the MNIST dataset. For training purposes, we employed model (1) with eight hidden layers, each comprised of 400 nodes. The training configuration, encompassing the choice of loss function, optimizer, and maximum number of training epochs, remained consistent with the parameters utilized in our MNIST experiments. The training process culminated in a remarkable training dataset accuracy of 99.39%, while the test dataset accuracy reached 53.59%.

In the initial phase of our experimentation, we computed the Hamming distance between a selected data point and a reference data point, maintaining a predefined fixed Euclidean distance of 0.1. These data points were sourced from the training, test, and adversarial datasets. It's essential to note that the adversarial dataset was generated using the same methodology applied to the MNIST dataset. Furthermore, the training dataset consisted of 10,000 data points, aligning with the identical number of data points present in the test and adversarial datasets.

The computed mean Hamming distances across the three datasets reveal values of 109.47, 130.19, and 142.80, accompanied by respective standard deviations of 40.44, 50.66, and 63.42 for the 10,000 data point pairs. Notably, these results diverge from our MNIST experiments, as they indicate a notable dissimilarity in the average Hamming distance between training data points and their corresponding samples compared to that observed in the test dataset. This discrepancy can be attributed to the suboptimal generalization of the trained neural network when applied to the test dataset, resulting in disparate Hamming distance profiles between the training and test datasets.

It's important to underscore the significance of Hamming distance as a lower boundary for quantifying the number of polyhedral boundaries traversed during the trajectory between two polyhedra. The variance in mean Hamming distances among the three datasets implies that, on average, a greater number of polyhedral boundaries exist between an adversarial dataset and its sampled data point.

Subsequently, we conducted a random sampling of 200 data points from the training, test, and adversarial datasets and computed the linear models of the polyhedra within which they resided. We then calculated the Chebyshev centers and their corresponding radii. The statistics concerning the radii of the polyhedra containing the sampled training, test, and adversarial data points are presented in Table 7. The results shed light on the distinctive nature of the average polyhedral sizes across the three datasets. Notably, the training data points exhibited the most substantial polyhedral size, followed by the test data points, with the adversarial data points displaying the smallest polyhedral size. This observation aligns with the findings derived from the Hamming distance measurements.

**Table 7:** Statistics of the radius of the largest inscribed ball within polyhedra: training, test, and adversarial datasets.

	**Mean**	**SD**	**Max**	**Min**
Train	0.038	0.015	0.081	0.015
Test	0.021	0.016	0.069	0.089
Adv	0.017	0.0095	0.051	0.0054

Similar to the obersvation on MNIST dataset, the reduced polyhedral size within the adversarial dataset accentuates the network's decision boundaries' susceptibility to manipulation and their propensity to undergo variations when exposed to adversarial inputs. These insights underscore the intricate interplay between geometric characteristics and network behavior, reinforcing the critical need for comprehensive and robust neural network assessments.

The analysis on MNIST and CIFAR datasets has revealed valuable insights into the performance and adaptability of trained neural networks. By employing a combination of Hamming distance and the Chebyshev center method, we are capable of gauging a network's generalization capability and its resilience to real-world data variations and adversarial challenges. These insights not only enhance our understanding of neural network behavior but also provide practical guidance for creating more robust and versatile neural systems capable of effectively navigating the complexities of real-world data and adversarial scenarios.

## 6. Conclusion

In this work, we present a toolbox for exploring aspects of the polyhedral decomposition (and other associated geometries) of neural networks. Our toolbox allows for the calculation of binary vectors, derivation of polyhedron linear models, extraction of active bits, and enumeration of neighboring polyhedra. Leveraging this toolbox, we investigate the geometric properties of neural networks using the Hamming distance, bisection method, and Chebyshev centers. Through implementation on toy datasets and the MNIST dataset, we validate the effectiveness of our approach and gain insights into the underlying geometries of neural networks. Overall, our work provides a contribution to the understanding and analysis of ReLU neural network structures, decompositions, and behaviors. This paper serves as a proof of concept, laying the foundation for future endeavors. Subsequent work will extend the application of the toolbox and methodologies to conduct comprehensive geometric analyses on much larger real-world datasets together with much more intricate neural network architectures. This includes enhancing model generalization, optimizing model structures, and exploring the design of novel network architectures.

## Data availability statement

The raw data supporting the conclusions of this article will be made available by the authors, without undue reservation.

## Author contributions

YL: Conceptualization, Formal analysis, Investigation, Methodology, Software, Supervision, Writing—original draft, Writing—review & editing. TC: Data curation, Formal analysis, Software, Writing—original draft. CP: Supervision, Writing—review & editing. MK: Funding acquisition, Project administration, Supervision, Writing—review & editing.
